# Hemodynamic and Metabolic Correlates of Perinatal White Matter Injury Severity

**DOI:** 10.1371/journal.pone.0082940

**Published:** 2013-12-11

**Authors:** Art Riddle, Jennifer Maire, Victor Cai, Thuan Nguyen, Xi Gong, Kelly Hansen, Marjorie R. Grafe, A. Roger Hohimer, Stephen A. Back

**Affiliations:** 1 Department of Pediatrics, Oregon Health & Science University, Portland, Oregon, United States of America; 2 Department of Public Health and Preventive Medicine, Oregon Health & Science University, Portland, Oregon, United States of America; 3 Department of Pathology, Oregon Health & Science University, Portland, Oregon, United States of America; 4 Department of Obstetrics and Gynecology, Oregon Health & Science University, Portland, Oregon, United States of America; 5 Department of Neurology, Oregon Health & Science University, Portland, Oregon, United States of America; Robert Debre Hospital, France

## Abstract

**Background and Purpose:**

Although the spectrum of perinatal white matter injury (WMI) in preterm infants is shifting from cystic encephalomalacia to milder forms of WMI, the factors that contribute to this changing spectrum are unclear. We hypothesized that the variability in WMI quantified by immunohistochemical markers of inflammation could be correlated with the severity of impaired blood oxygen, glucose and lactate.

**Methods:**

We employed a preterm fetal sheep model of in utero moderate hypoxemia and global severe but not complete cerebral ischemia that reproduces the spectrum of human WMI.

Since there is small but measurable residual brain blood flow during occlusion, we sought to determine if the metabolic state of the residual arterial blood was associated with severity of WMI. Near the conclusion of hypoxia-ischemia, we recorded cephalic arterial blood pressure, blood oxygen, glucose and lactate levels. To define the spectrum of WMI, an ordinal WMI rating scale was compared against an unbiased quantitative image analysis protocol that provided continuous histo-pathological outcome measures for astrogliosis and microgliosis derived from the entire white matter.

**Results:**

A spectrum of WMI was observed that ranged from diffuse non-necrotic lesions to more severe injury that comprised discrete foci of microscopic or macroscopic necrosis. Residual arterial pressure, oxygen content and blood glucose displayed a significant inverse association with WMI and lactate concentrations were directly related. Elevated glucose levels were the most significantly associated with less severe WMI.

**Conclusions:**

Our results suggest that under conditions of hypoxemia and severe cephalic hypotension, WMI severity measured using unbiased immunohistochemical measurements correlated with several physiologic parameters, including glucose, which may be a useful marker of fetal response to hypoxia or provide protection against energy failure and more severe WMI.

## Introduction

Hypoxia-ischemia (H-I) is a major cause of perinatal cerebral white matter injury (WMI) [1], the most common lesion in children with cerebral palsy (CP) [2]. Survivors of premature birth [3] and infants with congenital heart disease [4] have an increased risk for WMI and its associated non-progressive motor deficits and cognitive/learning disabilities. Although advances in the care of critically ill newborns have coincided with a reduction in the severity of WMI as defined by neuropathology [5] and MRI studies [6], considerable variability in the spectrum of WMI persists. Advances in neuro-imaging have identified a shift from previously common large necrotic lesions (periventricular leukomalacia; PVL) to less severe focal or diffuse non-necrotic WMI [6]. The latter forms of WMI occur in association with discrete foci of microscopic necrosis [7], which continue to have a high incidence, but comprise a minor component of the total burden of human WMI [5]. We have observed a similar spectrum of chronic WMI, as defined by high field ex vivo MRI studies in an instrumented preterm fetal sheep preparation where diffuse non-necrotic WMI predominates [8].

Since clinical sequelae are greater for children with more severe WMI [3], there is a critical need to define the pathophysiological mechanisms that contribute to variability in the severity of WMI. Progress to define these mechanisms has been hampered by the lack of histopathological outcome measures that allow the broad range of WMI severity to be defined. Although prior studies in preclinical animal models sought to define pathophysiological predictors of fetal brain injury [9], the full spectrum of contemporary WMI was not recognized at that time. WMI was also not analyzed using unbiased quantitative approaches. Recently, we developed objective methods to quantify the magnitude of astroglial and microglia reactivity in chronic WMI [5,8]. This approach provides continuous predictive outcome measures of the severity of WMI. These cellular responses to WMI were found to be more diffuse and greater in magnitude than supported by conventional histopathological approaches. We hypothesized that these unbiased measures are more sensitive to mild and intermediate levels of WMI and therefore allow a stronger association with continuous physiological variables and indices of WMI.

Here, we applied these quantitative histopathological measures to determine how the severity of WMI following H-I was related to residual cephalic blood pressure, arterial blood oxygen content (CaO_2_), and blood glucose or lactate concentrations. We employed a model of fetal ovine global cerebral ischemia that generates a broad spectrum of WMI that is similar to human: diffuse non-necrotic WMI predominates and the burden of microscopic and macroscopic necrosis is low [10,11]. The severity of WMI was significantly associated with the magnitude of the residual perfusion pressure and oxygen content measured at the end of 25 min of H-I when measured by unbiased approaches, but not traditional neuropathology. Surprisingly, blood glucose level was the factor most significantly associated with WMI severity, suggesting that glucose may be a useful indicator of the fetal response to hypoxia or may even modulate the burden of necrotic WMI during periods of severely impaired oxygenation. 

## Materials and Methods

### Animal Surgery

All studies were performed in a core facility of the OHSU Department of Comparative Medicine strictly adhering to a protocol approved by the OHSU IACUC (Protocol A688). Sterile surgery was performed on time-bred, twin pregnant sheep of mixed western breed between 88–91d of gestation (term 145d) as previously described [10]. For detailed methods on surgical procedures, physiological monitoring and blood analysis, see 11,12. In brief, a catheter was non-occlusively placed in the carotid artery, another in the amniotic fluid and an inflatable silastic occluder (4 mm; In Vivo Metrics, Healdsburg CA) was placed on the brachiocephalic artery (BCA). The arterial catheter tip was distal to the occluder. The occipital vertebral arteries were bilaterally ligated. Uninstrumented twin fetuses were used as controls for histopathological assessments. No evidence of twin-twin transfusion syndrome, including marked fetal discordance, fetal anemia, polycythemia, oligohydramnios, or polyhydramnios was found. 

### Cerebral Hypoxia-Ischemia Studies and Physiological Monitoring

On the third or fourth post-operative day using methods similar to that previously reported [11], pressure transducers and a digital chart recorder (PowerLab 16/30, ADInstruments, Sydney, Australia) recorded pressure in the fetal carotid artery relative to amniotic fluid pressure. Fetal heart rate (FHR) was calculated from triplicate measurements of the arterial pressure pulse intervals over a continuous recording of no less than 20 seconds. An arterial blood sample was taken following a 5-minute baseline period during which arterial blood pressure (BP) was measured relative to amniotic fluid pressure and fetal heart rate (FHR). Thereafter, moderate maternal and fetal hypoxemia was caused by lowering the inspired oxygen fraction of the ewe to 10.5%. After 5 min of hypoxia a blood sample was collected and sustained cerebral hypoperfusion was initiated for 25 min by occlusion of the common BCA. Residual BP was measured and another arterial blood sample was anaerobically collected. At 10 min after the conclusion of hypoxia-ischemia, pressures were measured and a final arterial blood sample was taken. Table 1 contains the average values of these blood based parameters under four conditions: baseline, 5 minutes of hypoxia, 25 minutes of hypoxia and cephalic occlusion, and after a 10 minute “recovery” period.

**Table 1:** Summary of sheep physiological responses.

		Baseline	Hypoxia	Hypoxia-Ischemia	Recovery
BP	(mmHg)	34.2 ± 3.3	32.4 ± 3.6	7.1 ± 2.2*	33.0 ± 3.3
HR	(b/min)	202 ± 15	228 ± 36*	NA	217 ± 21
pHa		7.39 ± 0.02	7.41 ± 0.02	7.35 ± 0.03*	7.31 ± 0.04*
PCO_2_	(torr)	47.6 ± 3.2	44.6 ± 2.9*	45.7 ± 3.2	47.8 ± 2.9
PO_2_	(torr)	25.0 ± 2.6	16.0 ± 3.3*	19.3 ± 4.6*	27.8 ± 2.6
Hb	(g/dl)	9.2 ± 0.8	9.2 ± 0.7	10.4 ± 0.9*	9.7 ± 0.9
SatO_2_	(%)	66.4 ± 5.7	35.0 ± 12.5	45.4 ± 15.4	69.4 ± 5.6
Hct	(%)	28.6 ± 2.2	28.7 ± 2.2	32.1 ± 2.8	29.9 ± 2.8
CaO_2_	(ml/dl)	8.2 ± 0.9	4.3 ± 1.6*	6.0 ± 2.3*	8.3 ± 0.8
Glucose	(mM)	1.3 ± 0.2	1.2 ± 0.2	1.7 ± 0.2*	1.6 ± 0.2
Lactate	(mM)	1.4 ± 0.3	1.5 ± 0.3	3.6 ± 0.6*	4.7 ± 0.6*

Physiological responses to H-I. Mean ± standard deviation. *, p < 0.05 vs. baseline (ANOVA or ANOVA on Ranks if variances were unequal. Post hoc testing was Dunnet's or Dunn’s testing as appropriate. NA indicates not available.

### Blood analysis

Blood samples were analyzed for arterial pHa, PaO_2_, PaCO_2_ corrected to 39.5°C, hemoglobin content (Thb), arterial oxygen saturation (SatO_2_) and hematocrit (Hct), glucose (Glu), lactate (Lac) and arterial oxygen content (CaO_2_; ABL725 blood gas analyzer, Radiometer Medical A/S, Bronshoj, Denmark). 

### Tissue collection and processing

The ewe and fetuses were euthanized (Euthasol) at 1 week (*n* = 10) or 2 weeks (*n* = 7) following completion of the occlusion protocol. Fetal brains were divided mid-sagitally and cut into five equivalent coronal blocks (6-10 mm thick) in proportion to the distance between the frontal and occipital poles. Tissue blocks were immersion fixed at 4°C in 4% paraformaldehyde in 0.1M phosphate buffer (pH 7.4) for 3-5 days and then stored in phosphate-buffered saline at 4°C.

### Immunohistochemistry

Tissue blocks were split coronally and dedicated to paraffin and free-floating preparations. Free-floating blocks were serially sectioned at 50 μm using a Leica VTS1000 vibrating microtome (Leica Microsystems Inc., Bannockburn, IL). The detailed immunohistochemical protocols to visualize GFAP-labeled astrocytes and Iba1-labeled microglia were performed as previously described [8,10]. Astroglia were visualized with rabbit glial fibrillary acidic protein (GFAP) antisera (1:500, Z-0334; DAKO, Carpinteria, CA). Microglia and macrophages were visualized with a rabbit anti-ionized calcium-binding adapter molecule 1 (Iba-1) antibody (1:500; 019-19741; Wako Chemicals, Richmond, VA). Appropriate anti-mouse and anti-rabbit AlexaFluor secondary antibodies were used for visualization of primary antibodies (1:500; Invitrogen, Carlsbad, CA). Tissue sections were counterstained with Hoechst 33342 (Invitrogen/Life Technologies). 

### Quantification of GFAP or Iba1 stained area fraction

WMI was assessed in experimental animals (n = 17) and in their twin controls by an unbiased approach where no lesions were selected. Hence, the image analysis data reflects the overall response of astrocytes and microglia across the entire white matter ROI. The validity of this approach is supported by our recent findings that both astroglial and microglial responses to injury occur diffusely across human white matter and extend beyond the lesion boundaries defined by hematoxylin and eosin (H & E) [5]. All frontal cerebral white matter dorsal to a horizontal tangent to the lateral ventricle at the level of the head of the caudate nucleus was analyzed by a single blinded investigator (VC). Using a motorized x,y stage mounted on an inverted fluorescent microscope, montages were generated from triplicate tissue sections that were alternately stained for GFAP or Iba-1. Montages were captured at 5x magnification (DMIRE2 inverted fluorescent microscope, Leica Microsystems Inc.) using an Orca ER cooled CCD camera (Hammamatsu Photonics, Hammamatsu, Japan) and Stereo Investigator (MicroBrightField Inc., Williston, VT). To quantify immunohistochemical staining, a pixel-intensity histogram was generated for this region using ImageJ (NIH; rsbweb.nih.gov/ij/index.html) [5,13] and exported to a spreadsheet. The peak of the histogram was calculated using the three highest frequency bins, and the histogram curve integrated towards the background pixel side of the peak and a value obtained for the area of this region. This area was then doubled to estimate the total distribution of background voxels in the image. The total background area was subtracted from the total region area to define the GFAP-labeled area. 

### Histopathological Analysis of Injury by Ordinal Rating Scale

Paraffin-embedded tissue sections (6 µm) from the adjacent tissue blocks to those stained for GFAP and Iba1 were stained in triplicate for H & E. We employed an ordinal WMI rating scale to define the burden of WMI. The percentage of the white matter with necrosis was scored by a blinded neuropathologist (MG) as follows: 0, no necrosis; 1, 1-25%; 2, 26-50%; 3, 51-75% and 4, 76-100%.

### Statistical Methods

Data analysis was performed using SAS software 9.2 (SAS Institute Inc., Cary, NC, USA) and R software version 2.15.3 Necessary assumptions (e.g., normality, equal variance) were examined before statistical analyses were performed. Comparisons between baseline and recovery physiologic measures and control and experimental GFAP and Iba1 area fractions were made using ANOVA with least significant difference post hoc testing. The following studies tested for an association across the entire study group using a linear mixed effects model (LME) [14]: Iba1 area fraction vs. GFAP area fraction; measures of Iba1 area fraction or GFAP area fraction relative to cephalic pressure, CaO_2_, blood glucose and blood lactate levels adjusted for the effect of survival time on the sample population (i.e., week 1 or week 2 survival after ischemia). Regression analysis by ANCOVA was used to define the correlation between CaO_2_, blood lactate and blood glucose adjusted for the effect of survival time (i.e., week 1 or week 2 survival). Regression analysis by ANCOVA also defined the relative contributions of MABP, CaO_2_ and blood glucose to WMI severity, adjusted for the effect of survival time, as quantified from the averaged area fraction of GFAP and Iba1 staining in the white matter. Survival time was not determined to be a significant contributor to outcomes in any tests performed. All statistical tests were considered significant at a p < 0.05 level of error. Physiologic data are presented as mean ± standard deviation.

## Results

### WMI severity defined by unbiased quantification of astroglial and microglial reactivity

**Figure 1:**
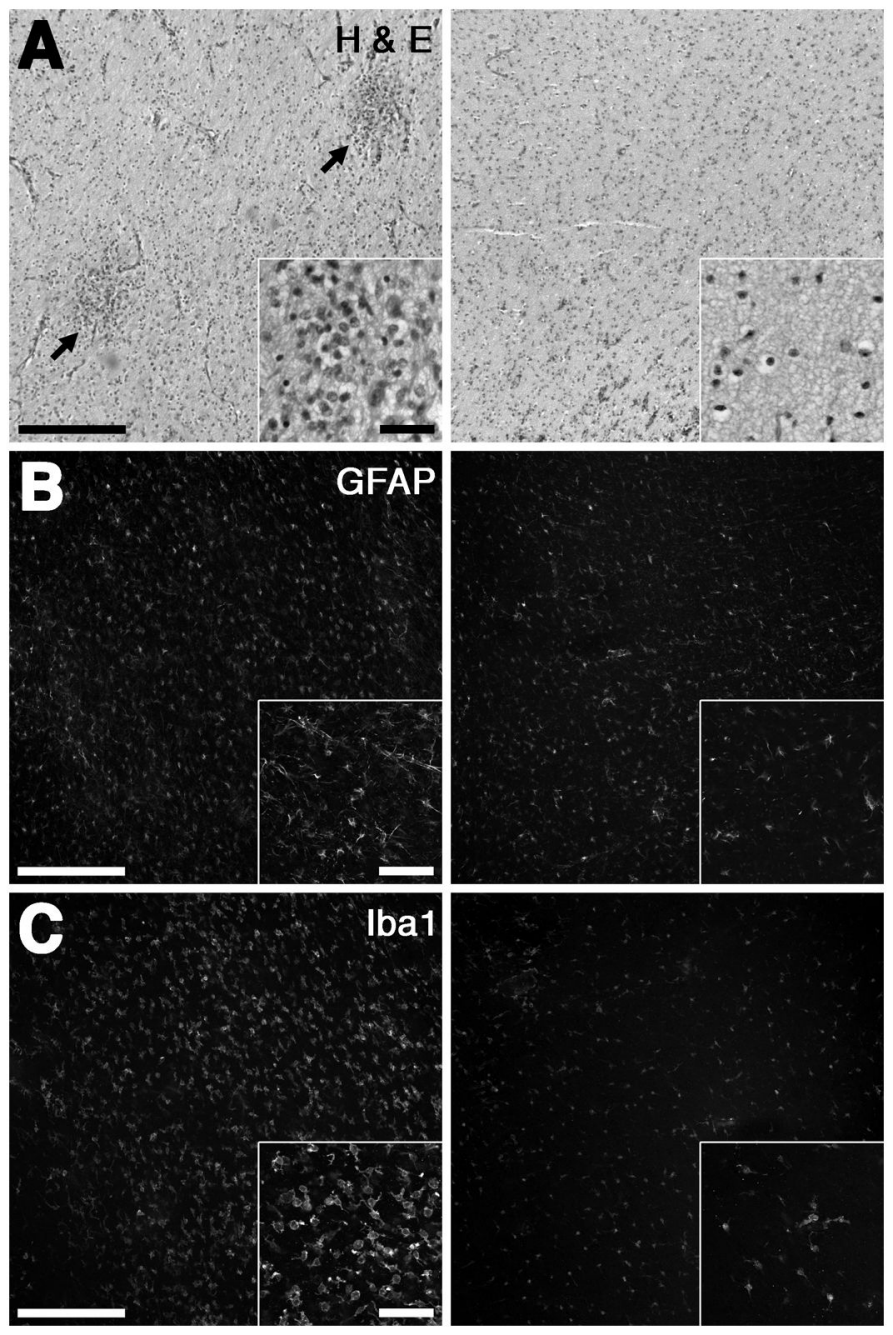
Spectrum of injury in H-I treated animals. Spectrum of WMI varied from more severe (left panels) to more mild (right panels). In more severe animals, H & E staining showed areas of focal necrosis with increased cellularity (A, left, arrows), filled with apparent dark pyknotic nuclei (inset). Many H-I treated animals appeared normal by H & E (A, right). Diffuse intense GFAP (B, left) and Iba1 (C, left) reactivity was visualized in the WM of animals with H & E evidence of focal necrosis. Animals with WM that appeared normal by H & E had evidence of diffuse mild reactivity on GFAP (B, right) and Iba1 (C, right) staining. Scale bars; main panels, 200 µm; inset panels 40 µm.

Despite the broad range of WMI observed in human autopsy cases [7], there is no consensus on the optimal approach to objectively quantify the severity of WMI observed in response to preterm H-I. In this fetal ovine study, the spectrum of WMI observed on H & E staining ranged from normal appearing white matter to focal areas of necrosis with increased cellularity and pyknotic nuclei (Figure 1A). Unexpectedly, white matter that appeared normal by H & E (Figure 1A, right) displayed evidence of mild cellular activation as visualized by staining for GFAP (Figure 1B, right) and Iba1 (Figure 1C, right). Lesions with focal necrosis on H & E, displayed GFAP and Iba1 staining that was diffusely increased (Figure 1 B, C, left, respectively). 

**Figure 2:**
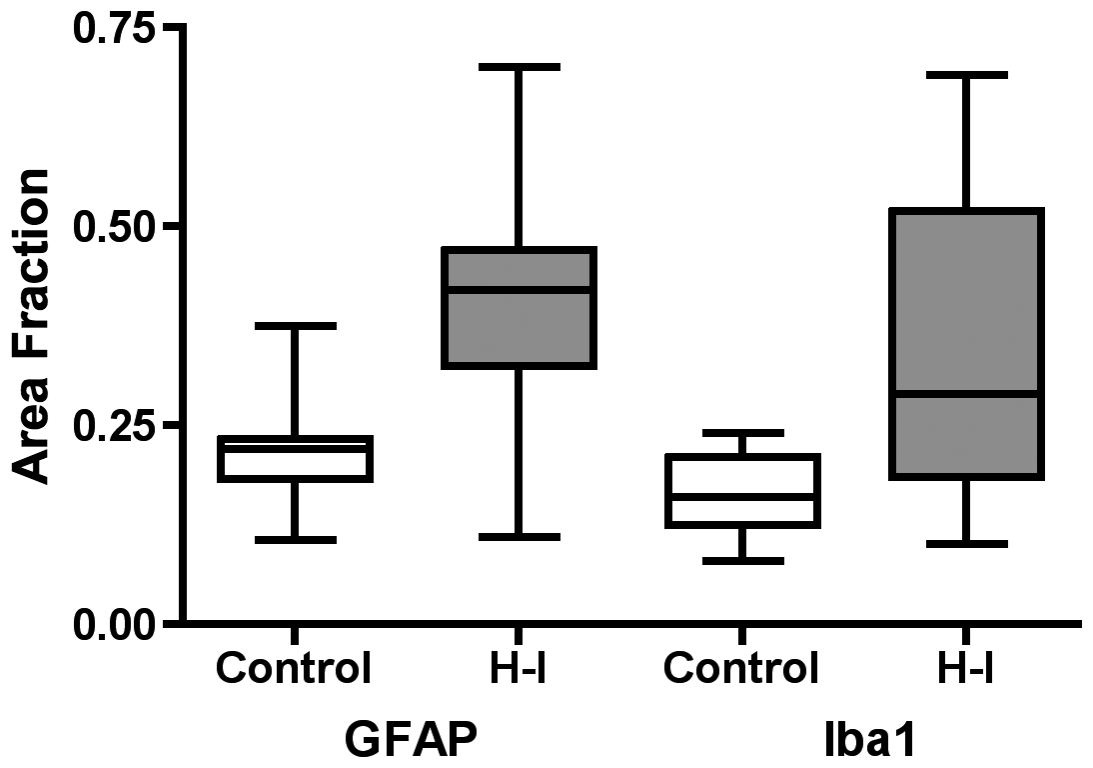
GFAP and Iba1 are significantly elevated in animals treated **with H-I**. Area fraction measures for twin sham and H-I treated animals depicting maximum, 75^th^ quartile, median, 25^th^ quartile, and minimum. GFAP and Iba1 were both significantly increased in the H-I treated groups (p < 0.0001 and p < 0.001, respectively). In the H-I group, GFAP area fraction measures were elevated above the range of control values in more animals than Iba1.

**Figure 3:**
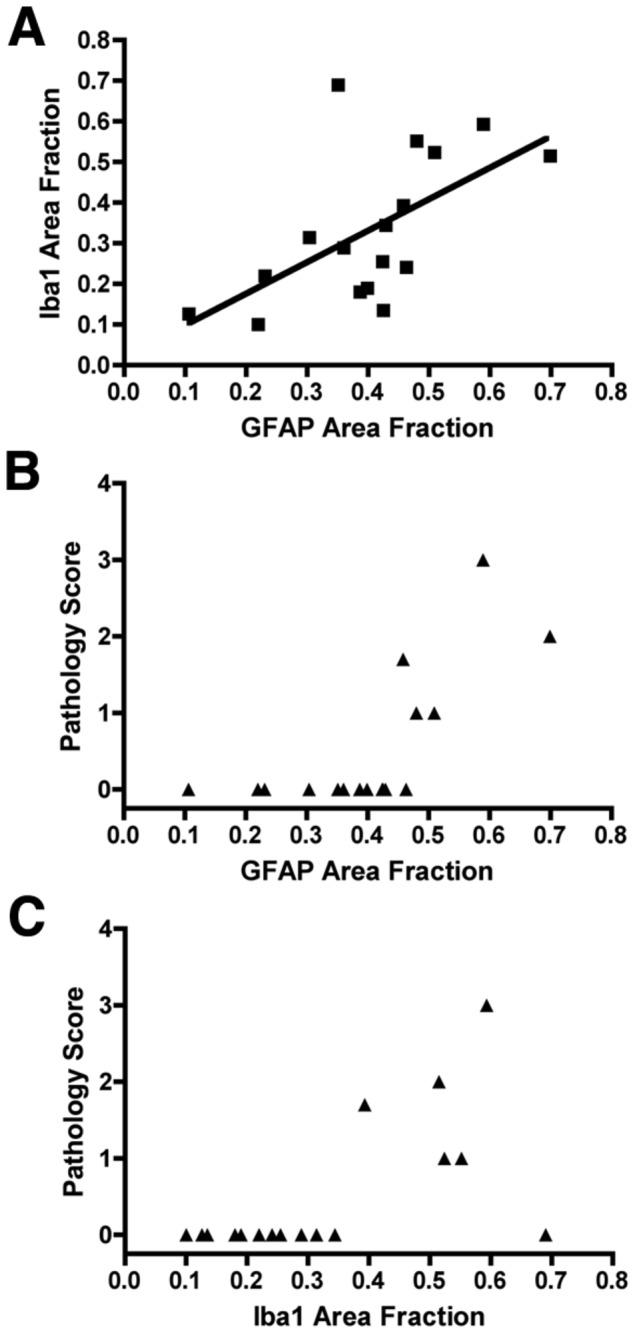
Unbiased markers of WMI are more sensitive to WMI than routine pathological analysis. A. Unbiased area fraction measures of GFAP and Iba1 correlated linearly across the range of WMI observed after H-I (p < 0.003; LME). B, C. Pathology rating of cases by a blinded pathologist did not correlate linearly with GFAP or Iba1 and failed to detect any injury (score of 0) in 12/17 cases that displayed a broad range of GFAP and Iba1 values.

To define the spectrum of WMI in the 17 animals that sustained global H-I, an ordinal rating scale was designed, based upon neuro-pathological review of H & E stained tissue sections, that estimated the burden of necrosis (see Materials and Methods). This scale was compared against an unbiased quantitative image analysis protocol that provided continuous histo-pathological outcome measures for reactive astrogliosis and microgliosis. We quantified the area fraction of the white matter that was stained for GFAP-labeled astrocytes or Iba1-labeled microglia. As previously reported [8], the levels of both GFAP and Iba1 were significantly elevated in H-I vs. twin brief hypoxia-only controls, (GFAP; 0.40 ± .40; vs. 0.22 ± 0.07, p < 0.0001, Iba1; 0.33 ± 0.18 vs. 0.16 ± 0.05, p < 0.001, ANOVA, Figure 2). The hypoxia-only group had GFAP and Iba1 values that did not differ from previously published values obtained from control animals not exposed to hypoxia[8]. Experimental GFAP measures are presented hereafter, but similar results were found for Iba1. Figure 3A demonstrates that unbiased estimates of WMI defined by quantification of GFAP and Iba1 were significantly correlated across a broad range of values (p < 0.003; LME). By contrast, WMI defined by our ordinal rating scale for WMI pathology, was not significantly associated with either the GFAP area fraction (Figure 3B) or the Iba1 area fraction (Figure 3C). Although 12 of the 17 cases defined by H & E had a pathology score of zero (i.e., no apparent injury), these cases displayed a broad range of WMI, as defined by the GFAP or Iba1 area fraction that encompassed approximately 2/3 of the range of area fraction values in our cohort. Thus, these quantitative measures of reactive glia provided a robust measure of a broad range of WMI that was more sensitive to lower levels of WMI that appeared uninjured by routine pathological analysis.

### H-I results in significant fetal metabolic stress

**Figure 4:**
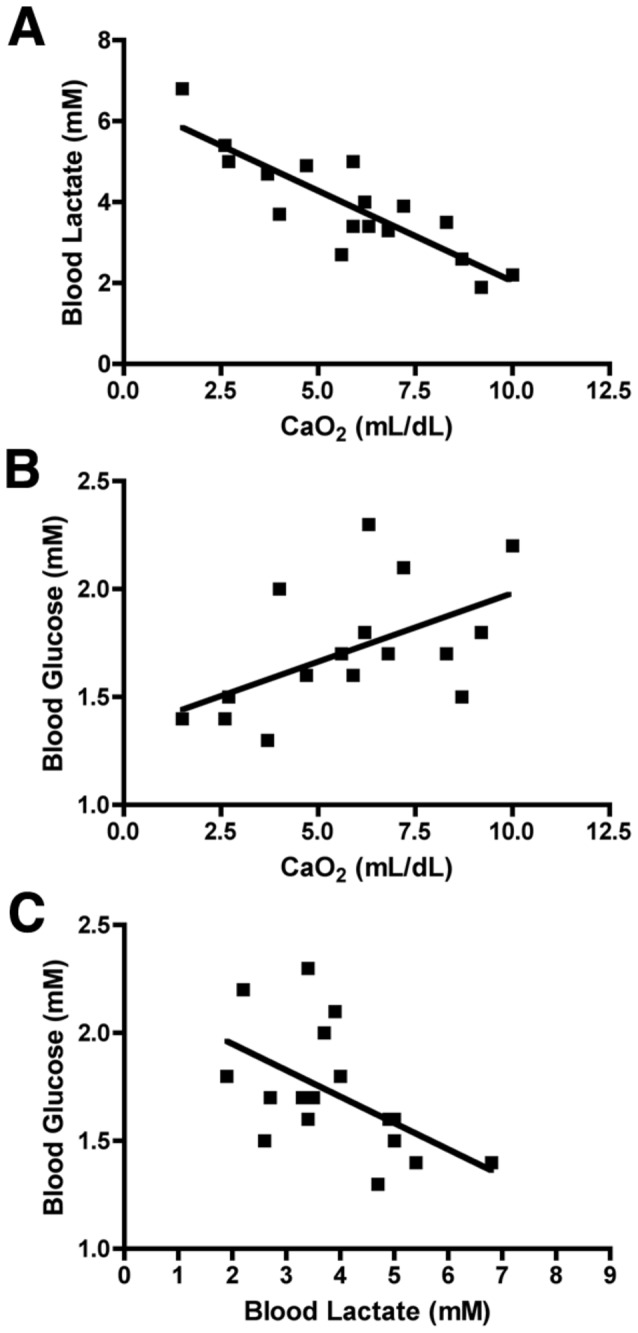
Markers of metabolic stress correlate with degree of hypoxemia. A; Blood lactate levels were strongly negatively correlated with CaO_2_ (r^2^ = 0.74; p < 0.0006, ANCOVA). B; Blood glucose also showed a strong trend toward positive correlation with CaO_2_ (r^2^ = 0.30; p = 0.07). C; Blood Glucose and blood lactate also showed a strong trend toward an inverse relationship (r^2^ = 0.30; p = 0.06), such that lactic acidosis was associated with declining glucose levels.

To define the response to H-I, we first analyzed blood lactate levels as a marker of metabolic stress. In response to H-I, lactate levels were very significantly correlated with CaO_2_ Figure 4A; r^2^ = 0.74; p = 0.0006; ANCOVA). There was, thus, a highly significant association between the magnitude of hypoxemia and lactic acidosis. This range of metabolic stress was also reflected in the blood glucose levels detected in the setting of hypoxemia (Figure 4B; r^2^ = 0.30; p = 0.07; ANCOVA). Glucose levels were directly correlated with CaO_2_, consistent with the notion that hypoxemia was related to enhanced glucose consumption. An inverse relationship between blood glucose levels and lactate levels was observed that supported that lactic acidosis was associated with declining glucose levels (Fig, 4C; r^2^ = 0.30; p = 0.06; ANCOVA). No association was found between baseline physiologic measures and WMI (not shown). Thus, the variable degree of hypoxemia observed in our model was associated with metabolic stress as measured by increased blood lactate levels and decreased blood glucose concentrations.

### Cephalic blood pressure and *C*aO_2_ are significantly associated with WMI severity

**Figure 5:**
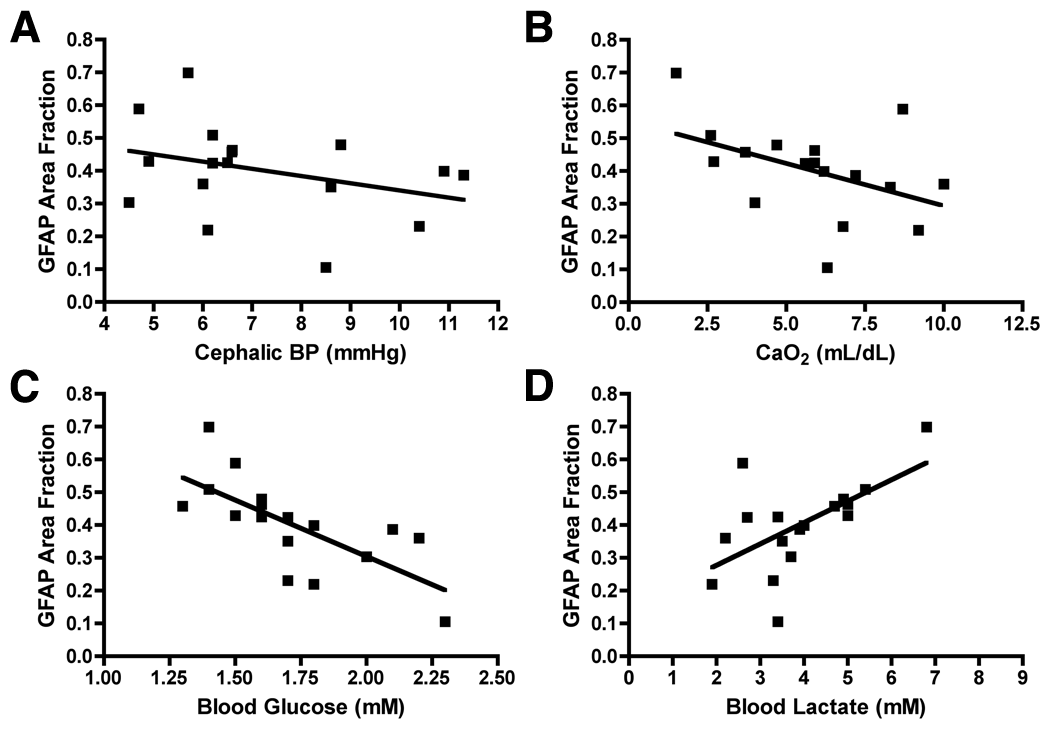
Physiologic and metabolic markers are associated with unbiased markers of WMI severity. A; Measured at the end of H-I, cephalic BP showed a significant inverse linear relationship with WMI measured by GFAP area fraction (p < 0.04, LME). B; CaO_2_ also was significantly associated with WMI (p < 0.02, LME). C; Unexpectedly, blood glucose was the most significantly and negatively associated with WMI, (p < 0.0001, LME). Increased serum lactate showed a significant positive linear association with WMI (p < 0.0001; LME).

Infants who sustain persistent hypotension display an enhanced risk for WMI. We found a significant negative association between the cephalic BP measured near the end of ischemia and the severity of WMI as measured by GFAP (Figure 5A; p < 0.04), and Iba1 staining intensity (not shown; p < 0.006; LME). To define the contribution of hypoxemia to the severity of WMI, we analyzed the association between the CaO_2_, measured just before the end ischemia, and the magnitude of WMI, as measured by GFAP (Figure 5B) and Iba1 (not shown). The CaO_2_ was similar to cephalic BP as a significant hemodynamic correlate of WMI (p < 0.02 for GFAP; p = 0.0013 for Iba1; LME). 

### Arterial blood glucose is strongly correlated with WMI severity

We next examined the association between arterial blood glucose levels and WMI. The normal range for basal glucose levels for the study was 1.4 ± 0.2 mM. During H-I, there was a significant increase in blood glucose to 1.7 ± 0.3 mM (p < 0.01). We observed a very significant negative association between the blood glucose levels during the period of ischemia and the severity of WMI as measured by GFAP (Figure 5C; p < 0.0001; LME) and Iba1 (not shown; p < 0.0001; LME). Hence, higher blood glucose levels during ischemia were associated with less severe WMI. Moreover, serum lactate levels during ischemia were positively associated with the severity of WMI as measured by GFAP (Figure 5D; p < 0.0001; LME) and Iba1 (not shown; p < 0.0001; LME). Hence, an elevation in lactate levels was significantly associated with more severe WMI, and higher blood glucose levels during ischemia were associated with less severe WMI.

**Figure 6:**
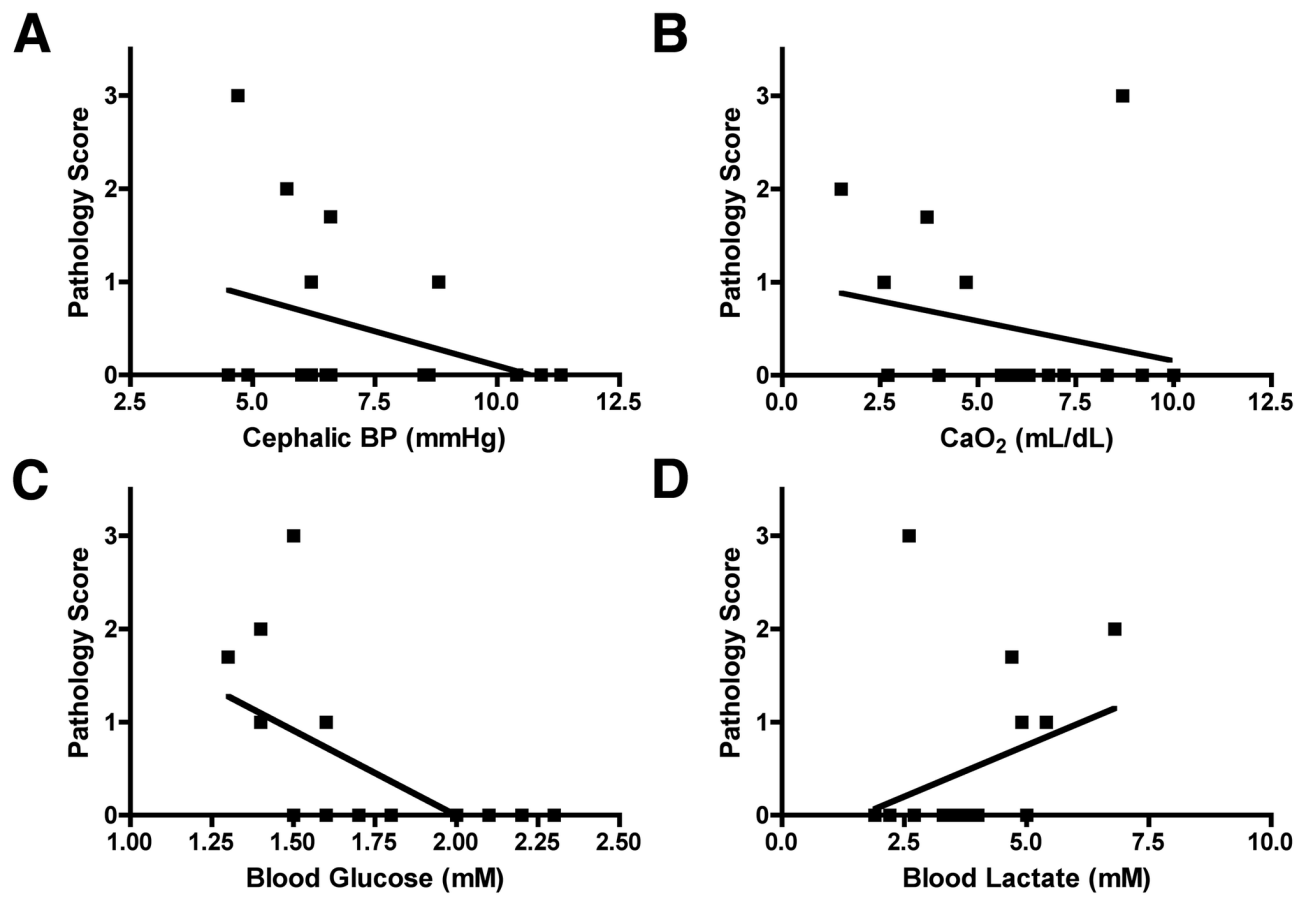
Physiologic and metabolic markers are not associated with an ordinal pathological rating scale. Cephalic BP, A, CaO_2_, B, blood glucose, C, and blood lactate, D, compared with our ordinal pathology rating scale as a measure of WMI. The pathology rating scale did not follow a normal distribution and was not linearly associated with physiologic or metabolic markers that showed significant association with unbiased markers of WMI.

We next analyzed cephalic BP (Figure 6A), arterial CaO_2_ (Figure 6B), blood glucose (Figure 6C) and blood lactate (Figure 6D) using our ordinal pathology rating scale as the measure of WMI. For all of these variables, the pathology scores did not follow a normal distribution, which supported the relative insensitivity of this approach to correlate WMI with physiologic or metabolic variables.

### Relative contributions of physiological factors to the severity of WMI

**Figure 7:**
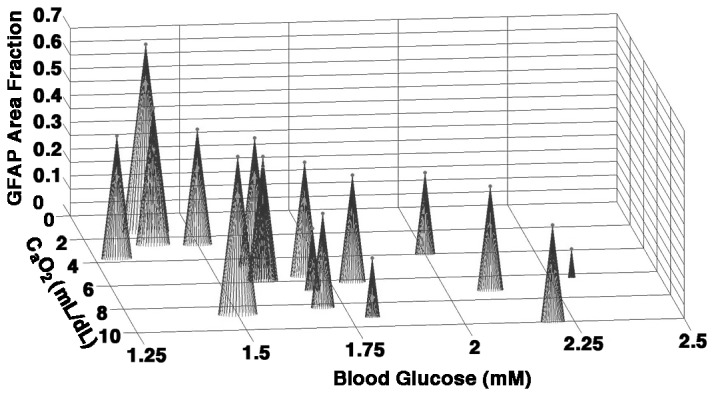
WMI severity is associated with the combination of blood glucose and CaO_2_. Those animals with the greatest degree of WMI (analyzed by GFAP area fraction) showed a combination of reduced CaO_2_ and blood glucose while those animals with less severe WMI had higher CaO_2_ and blood glucose.

We next analyzed the relative contributions of cephalic BP, CaO_2_ and blood glucose to WMI severity as quantified from the area fraction of GFAP and Iba1 staining in the white matter by multivariate regression analysis. Both WMI markers were strongly correlated. Blood glucose was the strongest predictor of WMI severity (r^2^ = 0.499 for GFAP; r^2^ = 0.298 for Iba1; ANCOVA), whereas cephalic BP (r^2^ = 0.15 for GFAP; r^2^ = 0.057 for Iba1) and CaO_2_ (r^2^ = 0.21 for GFAP; r^2^ = 0.08 for Iba1) were less strongly correlated to WMI severity. Figure 7 demonstrates the relationship of WMI severity relative to blood glucose levels and CaO_2_. The most severe WMI was associated with that cluster of animals that displayed the combination of lower blood glucose levels and lower CaO_2_, whereas higher blood glucose levels and CaO_2_ appeared to be associated with less severe WMI.

## Discussion

Definition of the pathophysiological factors that modulate the severity of perinatal WMI may provide the basis for improved interventions to reduce the chronic neurological morbidity associated with cerebral palsy in preterm survivors. Critically ill preterm neonates are at increased risk for WMI. In complete ischemia, the duration of the absence of flow is the critical factor that defines the magnitude of brain injury. However, in the preterm fetus complete cessation of flow does not usually occur [15]. Hence, we employed a model in which low residual cerebral blood flow (CBF) occurs during a fixed duration of BCA occlusion, and the levels of oxygen and glucose in the blood were analyzed for their relative contributions to the severity of WMI as measured by an unbiased index of WMI and an ordinal pathological rating scale. 

 The broad spectrum of WMI in preterm neonates suggested the hypothesis that fetal hypoxemia renders the white matter susceptible to more severe injury from global cerebral ischemia. In our model, occlusion of the BCA may be associated with increased placental perfusion and consequent increased fetal arterial oxygenation [9]. We have also found that during occlusion of the bilateral carotid arteries or BCA with OVA ligation there is a small but important residual blood flow (~10% of normal) [10], which importantly connects the H/I brain with the rest of the fetal body and placenta. In light of this residual perfusion, the interactions between peripheral and cerebral tissues should be considered as contributory to metabolic conditions leading to brain injury. The rise in oxygenation during isolated occlusion may deliver sufficient oxygen to cerebral white matter such that even a profound fall in CBF may not impair oxygen delivery enough to cause brain energy failure and WMI. Thus, we adapted our model of global cerebral ischemia by limiting systemic fetal oxygen delivery by reducing the mother's inspired oxygen concentration, which resulted in hypoxic hypoxia. Prior studies in preterm fetal sheep found that cerebral oxygen delivery was not maintained during hypoxic hypoxia [16], which suggested that important regulatory mechanisms are not fully developed in the immature brain that render it more vulnerable to hypoxic injury. In mid-gestation fetal sheep, major foci of cerebral white matter and subcortical gray matter necrosis developed when systemic hypotension was combined with hypoxia [17,18]. As concluded by Gunn [19], we also found no evidence of WMI in animals exposed to moderate hypoxia without cephalic ischemia or hypotension. This is also in agreement with previously published in age matched fetuses not exposed to hypoxia [8,11].

With this model of more consistent hypoxemia, we analyzed the relative contributions of several hemodynamic and metabolic factors that may play a role in the pathogenesis of more severe WMI and which are widely measured clinically. We defined associations between these factors using both a conventional histopathological injury rating scale and unbiased injury measures of astrogliosis and microglial activation. The values from pathology rating scales were not normally distributed and relatively insensitive to a wide range of more moderate WMI that was detected by an unbiased quantitative analysis that provided continuous histo-pathological outcome measures for astrogliosis and microgliosis derived from the entire white matter. Thus, commonly used pathological scales based on H&E are likely to underestimate moderate inflammatory WMI. Our results show that feasible and unbiased quantitative estimation of several white matter inflammation markers provide measures of WMI over a large dynamic range that are highly correlated with physiological predictors of brain injury, even across the narrow range of values present during H-I. 

Consistent with these findings, we found that fetal CaO_2_ was significantly correlated with the spectrum of WMI severity caused by cerebral ischemia and maternal hypoxia. Under normal physiological conditions in the near term fetus, when CaO_2_ decreases, the cerebral circulation responds by increasing CBF in order to maintain oxygen delivery to the brain. This cerebral vasodilatory hypoxic response is directly related to CaO_2_ [16,20]. Our data is consistent with the notion that there is a limit beyond which CBF cannot increase, which causes oxygen delivery to fall [21,22]. Although the brain responds by increasing oxygen extraction, our data suggests that under conditions of severe but incomplete ischemia, once the limit of oxygen extraction is reached, brain tissue hypoxia contributes to more severe WMI.

Although hypoglycemia occurs commonly in sick premature infants, its role in perinatal WMI has been unclear. Hypoglycemia can lead to long-term deficits that are associated with MRI-defined injury, especially to developing thalamus, parieto-occipital cortex, and associated white matter [23-25]. In our study, blood glucose was the factor most strongly associated with WMI severity and was modestly elevated above baseline in animals with milder WMI while animals with more severe WMI remained nearer baseline. The precise stimuli and mechanisms for glucose mobilization we found during fetal H-I remain uncertain. Fetal carotid chemoreceptors and baroreceptors are the primary mechanisms for increased plasma concentrations of catecholamines in preterm fetuses during H-I, which might act to increase glucose production from hepatic glycogen, perhaps working through glucagon and suppressed insulin [26,27]. Gluconeogenesis is less likely, especially in immature fetuses [28,29]. In late term fetuses these responses are mediated by cortisol, noradrenaline, adrenaline, arginine vasopressin and neuropeptide Y [28,30], but the roles of cortisol, NYP and VP are not well described in this preterm model. However, unexpectedly, those animals with less WMI, relatively higher BP, CaO_2_, and lower lactate, had the greatest increase in glucose in response to H-I, despite equivalent baseline glucose levels. Thus, animals with less severe WMI may have increased effectiveness or relative maturity of adaptive fetal responses, such as glucose release. The exact mechanisms by which these responses may mediate protection against H-I induced injury remain unclear and could be mediated by glucose itself or by other, as yet undefined means. 

Isolated hypoglycemia is associated with decreased cerebral glucose consumption but no change in cerebral O_2_ consumption [31,32]. Animal and human studies have shown that hypoglycemia in conjunction with H-I leads to more severe full term neonatal brain injury than H-I alone [33,34]. This may suggest that under conditions of limited oxygenation, ATP generation via glycolysis may be a critical determinant of WMI severity. During hypoxia-ischemia in human preterm infants, the cerebral effects of hypoglycemia may depend in part on the ability of the brain to utilize alternative oxidative substrates including ketone bodies, lactate, amino acids, and lipids [35]. Glucose is the principal energy source utilized in the fetal sheep [36-38]. During severe ischemia in near term fetal sheep, cerebral oxygen consumption falls in conjunction with a pronounced increase in glucose uptake [39]. Elevated blood glucose appears to help maintain electroencephalographic activity during ischemia in near term fetal sheep, perhaps by fueling additional anaerobic energy production [40]. Interestingly, glucose supplementation of cultures enriched in astrocytes was protective against severe in vitro hypoxic injury [41]. In near term hypoxic-ischemic piglets, glucose supplementation preserved brain ATP levels, but was associated with pronounced elevation in CSF lactate [42]. 

In our data, metabolic acidosis, as measured by fetal arterial blood lactate levels, was significantly and inversely associated with fetal blood oxygen levels and serum glucose. In brain tissue or CSF, we would expect increased anaerobic metabolism facilitated by increased glucose to generate higher brain lactate. However, as we did not measure brain or CSF lactate, we are unable to comment directly on any differences in the degree of anaerobic stress that may have occurred during our standardized H-I protocol. Arterial blood lactate sampled at the carotid is the venous admixture of blood from many peripheral organs including the brain. Within the intact animal, several additional metabolic processes that were not distinguished in this study affect blood lactate. Brain-derived lactate is partially trapped by the brain-blood barrier and brain lactate that reaches the circulation can be recovered back to pyruvate by either the placenta or the liver, which remain relatively better oxygenated than the upper body in this model. Hypoxia results in increased brain glucose uptake without an O_2_ uptake increase in preterm sheep [43]. This may be due to anaerobic metabolism and/or blood brain barrier based trapping of lactate in the brain [39,44]. Increased lactate clearance at the placenta is also a potential explanation for the observed inverse correlation of arterial lactate levels with plasma glucose [45,46]. Although most studies on maternal lowered O_2_ fraction or reductions in fetal umbilical blood flow involved near term fetuses, one study of 25 minutes of cord occlusion in immature fetuses showed inverse relationships between plasma glucose levels and lactate in injured fetuses [47]. Nevertheless, the role of metabolic acidosis in the pathogenesis of preterm brain injury remains controversial. Although the neonatal brain and immature fetal sheep may use lactate as a significant metabolic fuel [48], the ability to metabolize lactate as an alternative energy source may be limited or compromised during hypoxemia and metabolic acidosis arising from ischemia [49,50]. Hydrogen and bicarbonate ions do not diffuse readily through membranes, and induction of acute metabolic acidosis or alkalosis has not been shown to change CBF or autoregulation [51,52]. More recently, a 2005 Cochrane Review concluded that there was insufficient evidence that infusion of sodium bicarbonate reduces morbidity and mortality rates in preterm infants with metabolic acidosis [53].

The responses of the preterm human brain to similar insults are unknown, as is the potential predictive utility of blood glucose or protective role of hyperglycemia. Future studies of WMI should consider unbiased estimates of WMI as pathological rating scales may underestimate mild and moderate WMI. Intriguingly, we found that those animals with higher blood glucose levels during ischemia had less severe WMI. This may suggest that under conditions of limited oxygen delivery, ATP generation via glycolysis may be a critical determinant of WMI severity. Alternatively, glucose may serve as marker for fetal adaptive responses to hypoxia that are protective by alternative mechanisms. The role of glucose in modulation of damage during H-I in young fetuses remains unclear [54]. Future in vitro and in vivo studies are needed to analyze the potential of glucose supplementation to mitigate WMI.
